# S100B Protein as a Post-traumatic Biomarker for Prediction of Brain Death in Association With Patient Outcomes

**DOI:** 10.5812/atr.8549

**Published:** 2013-08-01

**Authors:** Moslem Shakeri, Atta Mahdkhah, Farid Panahi

**Affiliations:** 1 Department of Neurosurgery, Tabriz University of Medical Sciences, Tabriz, IR Iran; 2 Neurosciences Reserch Center, Tabriz University of Medical Sciences, Tabriz, IR Iran

**Keywords:** Posttraumatic Brain Death, Predictor, Biomarkers, S100B Protein, Outcome

## Abstract

**Background:**

S100B is a calcium-binding protein, belonging to the S100 family proteins which are characterized by their high solubility and, currently, comprises 21 members which are expressed in a cell-specific manner. If we can predict the possibility of definite brain death after brain injury, we will rescue some organs of body to transplant proposes.

**Objectives:**

In this regard our study focused on the S100B protein value in predicting brain death after head trauma. In this study, the use of serum level of protein S100, 24 hours after trauma has been considered as a reliable index for predicting brain death.

**Patients and Methods:**

72 patients (50 male and 22 female) aged 5 - 80 years old (median 40 ± 17.72 years) with severe head traumas (GCS≤8) were recruited in this cross-sectional study. Glasgow Coma Scale (GCS) and computed tomography (CT) scan findings were recorded for all patients, and then a single 5mL blood sample was obtained from each patient on admission, after 48 hours and a week later or after brain death to determine the level of S100B protein.

**Results:**

Primary and the last GCS of patients had a predictive value in determining brain death (P < 0.0005), also there was a significant correlation between GCS and level of S100B protein. There was a significant correlation between CT scan findings and S100B protein only after 48 hours of trauma.

**Conclusions:**

Changes in S100B protein, especially the levels of this dimer 48 hours after trauma can be used as marker to predict brain death. Alongside other known prognostic factors such as age, GCS and diameters of the pupils, however, this factor individually can not conclusive predict the patient's clinical course and incidence of brain death. However, it is suitable to use GCS, CT scan, clinical symptoms and biomarkers together for a perfect prediction of brain death.

## 1. Introduction

Brain injury is the third most common cause of mortality in the world. In spite of progress in monitoring and imaging studies, definite and correct prediction of brain death after brain trauma is not possible. Many studies have been done to make definite predictions of brain death after trauma (1 - 3). Prediction of brain death will enable us to save body organs if transplantation is considered. (4). Brain death is evaluated using confirmatory tests (Table 1) however misdiagnosis is possible if drug intoxication, hypothermia and locked-in syndrome are not recognized (4). 

**Table 1.** Clinical Criteria for Brain Death

Coma
Absence of motor responses
Absence of pupillary responses to light and pupils at midposition with respect to dilatation (4 – 6 mm)
Absence of corneal reflexes
Absence of caloric responses
Absence of gag reflex
Absence of coughing in response to tracheal suctioning
Absence of sucking and rooting reflexes
Absence of respiratory drive at a PaCO_2_that is 60 mmHg or 20 mmHg above normal base-line values^a^
Interval Between Two Evaluations, According to Patient’s Age
Term to 2 m old, 48 h
> 2 m to 1 y old, 24 h
> 1 y to < 18 y old, 12 h
≥18 y old, interval optional
Confirmatory Tests
Term to 2 m old, 2 confirmatory tests
> 2 m to 1 y old, 1 confirmatory test
> 1 y to < 18 y old, optional
≥ 18 y old, optional

^a^PaCO_2_ denotes the partial pressure of arterial carbon dioxide

Although many scoring systems including Glasgow Coma Scale Score (GCSS) (5), physiologic scoring systems e.g. Revised Trauma Score (RTS) (6) and Trauma Scoring and Injury Severity Score (TRISS) (7), have already been developed for assessment of injuries, however they are less valuable in prediction of outcome in traumatic brain injury (4). To overcome the possibility of misdiagnoses of brain death on the base of clinical criteria, biomarkers have attracted the researchers’ attention (8). Some of these markers have been proposed for the monitoring of brain damage, e.g. Neuron Specific Enolase (NSE), Creatine Kinase isoenzyme BB, 14-3-3 protein, myelin basic protein, Tau protein, polyamines and S100B protein (9-13). One of the brain specific biomarkers found in the past decades is S100B, expressed and produced by astrocytes in vertebrate brains (14). It is one of the calcium-binding proteins characterized by their high solubility and includes 21 members with cell-specific expression. In the CNS, the astrocytes are the major cells producing S100B protein in gray matter, and oligodendrocytes are the predominant S100B in protein producing cells in white matter (15). S100B protein can be produced in other cells such as adipocytes, chondrocytes, lymphocytes, bone marrow and melanoma cells (16). These data lead to controversy about the S100B protein and its dependency to brain injury (17-21). All authors agree that increasing levels of S100B within 12 hours after traumatic brain injury and cardiac surgery may correlate well with a poor outcome (22-24).

## 2. Objectives

This study focuses on the level of serum S100B after brain injury at the time of admission, 48 hours and a week later, or when the patient is labeled as brain death, to predict outcome of patients after traumatic brain injury.

## 3. Patients and Materials

Seventy-two trauma patients in the age range of 5 to 80 years and GCSS ≤ 8 admitted to trauma and ICU wards of Imam Hospital were enrolled in this cross-sectional study, after ethics committee approval. All patients were examined within 6 hours of their admission. Those patients with severe abdominal and chest injuries, or limb fractures, those under the age of 5, those who died within 24 hours of their admission, and finally all of those with a history of trauma, resuscitation, acidosis, hypotension, hypoxia, neurological diseases and spinal cord injuries were excluded from the study. All selected patients were examined by a neurosurgery resident, unaware of their selection, on admission, discharge or death. On the base of their condition, the patients were sedated, intubated and/or mechanically ventilated. Surgical decompression of hematoma and induction of barbiturate coma were considered if needed. Also, the patients were treated for effective control of cerebral perfusion pressure according to neurointensive guidelines (25). Initial CT scans were performed for all patients and evaluated by a radiologist who was not aware of the patient's profile. The following variables were recorded for each of the patients by a junior resident; age, sex, kind of trauma (road traffic accidents, falls from heights and strife), a history of specific diseases, the time of referral and hospital admission, length of hospital stay, GCS at admission and discharge or death, status of pupils, types of treatment (surgery - conservative), and neural status during hospitalization (improved - brain death). A single 5mL blood sample was obtained via an intravenous catheter from each patient on admission (first blood sample was taken 2 ± 0.5 hours after admission), 48 hours later, a week later or after brain death to determine the level of S100B protein. Serum samples were centrifuged, separated and stored at -70 °C until the analysis, and once gathered, samples from all patients were analyzed by enzyme immunoassay technique with a high degree of sensitivity. Doses greater than 0.5 micrograms per liter were considered as elevated and those above 0.15 micrograms per liter were normal and the boundary between these two values was considered. All data are expressed as mean ± SD, percentage and frequency. Obtained data were analyzed by using one-way ANOVA, Chi-square test, non-parametric Wilcoxon technique, Pearson correlation test, Repeated measures design test and Kolmogorov-Smirnov test. In all investigated cases, Statistical significance was set at 0.05.

## 4. Results

Seventy-two patients (50 male and 22 female) aged 5-80 (median 40 ± 17.72 years) with head traumas were recruited in this study.

All patients were divided into two groups according to clinical manifestation:

a) The first group, termed the “improved group", consisted of those who survived during hospitalization and included 42 patients.

b) The second group included those who died during hospitalization and were themselves divided into two categories:

1) The first group died from non-cerebral reasons and included 14 patients.

2) The second included 16 patients who died because of brain death.

In 4 cases with eye trauma, assessment of the pupillary response was not possible. The correlation between pupillary response and S100B protein level at different stages was not significant.

The results of treatment in the three groups was as follows: Craniotomy in improved group was carried out on 6 cases (14.3%) and conservative therapy on 36 patients (87.5%). In the group with death due to non-cerebral reasons, craniotomy was done for 6 (42.9%) and conservative treatment for 8 patients (57.1%). There were 7 cases (46.7%) with craniotomy and 8 cases (53.3%) with conservative treatment in those with brain death, P = 0.008, and there was a significant difference in treatment between these three groups. In fact, we had more craniotomy in the group with brain death in comparison with the other groups. There was no significant difference between patients in their laboratory findings, except in WBCs (P=0.003). The first and the last GCS of the patients had a predictive value in determining brain death (P < 0.0005). Association between primary GCS and S100B protein levels at the first hour, 48 hours later and at the final measurement, showed a significant correlation, with P = 0.02, P = 0,007, P = 0.006, respectively. Thus, Pearson correlation coefficients were -0.23, -0.29 and -0.92, respectively which represented a negative association between GCS and protein S100B. Also, a Significant correlation was observed between final GCS and protein S100B level at first, 48 hours later and final measurement with P = 0.027, P<0.0005 and P < 0.0005, respectively. The Pearson correlation coefficient was -0.22, -0.4 and -0.41, which indicated a negative association between the final GCS and protein S100B levels. Comparison of brain CT scan findings based on Marshall Classification with levels of protein S100B measured at three different stages only indicated a significant difference in S100B levels 48 hours after trauma (P = 0.017) (Table 2). 

**Table 2.** S100B Protein Levels Compared With Brain CT Scan Grade for the Three Groups

	Mean ± SD	Mean ± SD	Mean ± SD	Mean ± SD	Mean ± SD	P value
**S100 first**	0.41 ± 1.09	0.64 ± 1.04	0.81 ± 10.38	0.72 ± 1.22	0.99 ± 0.40	0.352
**S100 after 48 hours**	0.40 ± 0.98	1 ± 0.56	0.94 ± 1.59	1.17 ± 1.95	1.41 ± 0.56	0.017
**S100 last**	0.48 ± 0.92	0.41 ± 0.75	0.64 ± 1.07	0.84 ± 1.25	0.97 ± 0.45	0.342

Findings in terms of protein S100B levels on admission, after 48 hours and the final protein level between the three groups based on the following table, revealed a significant difference between levels of protein S100B for these three time points with P < 0.0005 (Table 3). 

**Table 3.** Comparison of the Initial, 48 Hours Later and the Final Levels of S100B Protein Between the Three Groups

	All Patients, Mean ± SD	Improvement, Mean ± SD	Death, Mean ± SD	Brain Death, Mean ± SD	P value
**S100 first**	1.13 ± 0.6	0.99 ± 0.45	1.12 ± 0.36	1.01 ± 0.93	0.013
**S100 after 48 hours**	1.42 ± 0.81	1.04 ± 0.5	1.46 ± 0.51	2.36 ± 0.94	< 0.0005^a^
**S100 last**	1 ± 0.58	0.77 ± 0.4	1.08 ± 0.45	1.53 ± 0.73	< 0.0005^a^

^a^ P values were significant (P ≤ 0.05)

**Figure 1.**
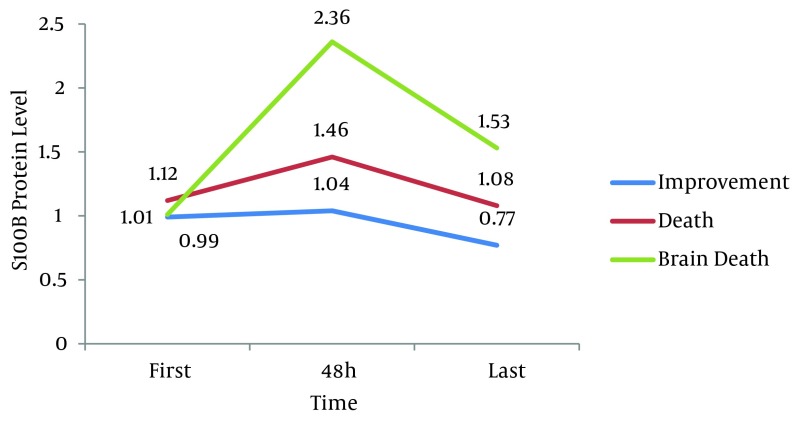
S100 Levels Between the Three Groups

Two by two Comparison of the groups showed (Figure 1) that the difference in the first and second blood sample was statistically significant (P < 0.0005), but the difference between step one and three was not significant (P > 0.05). 

## 5. Discussion

In this study, S100B protein level was assessed as a biomarker to predict brain death in patients with severe head trauma (GCS ≤ 8), and it was found that S100B protein levels increased in the brain death group, 48 hours after brain trauma. In 2008, Carlos et al. evaluated biological and methodological features of S100B. They noted that S100B protein is a marker of action or death of astrocytes and oligodendrocytes (26). Hayakata et al. noted that the level of S100B protein increased shortly after a head trauma, and then gradually decreased. In that study there was a significant correlation between increasing S100B protein and intracranial pressure (ICP) elevation (27). Another study in 2000 showed that the level of S100B protein increased shortly after the occurrence of trauma, in both the improved and brain death groups, but after 3 - 4 days, it normalized in the former group, but in the brain death group it normalized within 1 - 6 days and increased then after, for the progressive damage of brain cells (24). In our study ICP was not measured, however the results are different from those for the others. This may be due to the selection of patients with severe head injury. In our study the first blood sample was obtained 2 ± 0.5 hours after trauma. In a Meta-analysis in 2005 it was reported that highest sensitivity and specificity of S100B protein may be achieved if the first sample of blood which determines the level of S100B is obtained within 6 hours after trauma (28). In the present study patients with concomitant severe abdominal and chest injuries and limb fractures were excluded. It seems that skull fractures also may result in increased S100 level. In a study by Savola et al. it was shown that S100 level has a correlation with the severity of brain injury or in other words the severity of trauma. However, it was also found that massive extra-cranial trauma affects the serum levels of S100B protein (29). In the same direction, Unden et al. in their study in 2005 showed that S100 proteins can only be increased in 30% of patients with extremity fractures without brain damage (30). In our study, the level of S100B protein on admission, 48 hours later and at the final measurement was statistically and significantly different between the groups, and the level of S100B in the brain death group was higher than the others. Jang et al. noted the increased levels of S100B protein in patients who died within two weeks of their head trauma. The mean age of their patients was 34 years which is higher than the mean age of ours (mean age of 30.4 years) (31). The average of primary GCS score of patients in the current study was 5 ± 2. In the improved group it was 5.5 and in the brain death and non-cerebral death group, it was 3. Pelinka et al. studied serum S100 protein in patients with severe head traumas, without having multiple traumas. The average GCS of their patients on admission was 6. In this study, the use of serum level of protein S100, 24 hours after trauma has been considered a reliable index for predicting brain death (32). Nylen et al. in 2008 in Sweden measured the different dimmers of S100 in traumatic patients. The results suggest that all three dimmers of S100 protein increase in trauma patients and their amounts are different, but this difference was not statistically significant. Since measurement of the sub types of S100 protein was not cost effective compared with the S100B dimer (33), we preferred to measure only S100B dimer in our patients. In our study CT scan findings had a significant correlation with S100B protein only after 48 hours (P = 0.01). Oh et al. noted a significant correlation between MRI and CT scan findings and S100B proteins. There was a negative association between GCS and level of S100B protein; this shows that any patient with a low GCS will have a bad prognosis (34). Similar findings were reported by the Townend et al. study (35). In a prospective study in 2006 by Korfias et al. on 102 patients with severe head trauma, a correlation was found between increased levels of S100 protein and pupillary reaction, brain CT scan report and their improvement after a month.

Based on the results of this study, protein S100B changes and especially the levels of this dimer 48 hours after trauma can be used as a marker to predict brain death, along other known prognostic factors such as age, GCS and diameters of the pupils. However, this factor individually, cannot be conclusive for prediction of the patient's clinical course and brain death. Given the importance of S100B protein, accesses to facilities for its measurement, in hospitals managing brain trauma victims, seems to be necessary.
